# The Sustainability of Self‐Help Groups for Caregivers of Children With Disabilities in Kilifi, Kenya: The Changing Context of the COVID‐19 Pandemic

**DOI:** 10.1111/cch.70079

**Published:** 2025-04-12

**Authors:** K. Bunning, J. K. Gona, S. W. Wanjala, S. Hartley

**Affiliations:** ^1^ School of Health Sciences University of East Anglia Norwich UK; ^2^ Walezi Foundation Kenya Malindi Kenya; ^3^ School of Humanities and Social Sciences, Department of Social Sciences Pwani University Kilifi Kenya; ^4^ Department of Public Health and Primary Care, Faculty of Medicine and Health Sciences Ghent University Ghent Belgium

**Keywords:** caregivers, changing context, children with disabilities, COVID‐19 pandemic, low–middle income, quality of life, self‐help groups

## Abstract

**Background:**

Self‐help groups offer an approach to empowering the lives of caregivers and their children with disabilities in settings of limited resources and support. A study was conducted over a 5‐year period (2018–23) to assess the sustainability of 11 self‐help groups in Kilifi, Kenya, during which there was the COVID‐19 pandemic.

**Methods:**

An integrated framework of action research and mixed methods was carried out over three stages. Stage 1: pre‐pandemic, three self‐help groups participated in focus group discussions. Template analysis structured around the five pillars of the WHO community‐based rehabilitation matrix (CBR: health, education, livelihood, social, empowerment) was carried out. Stage 2: inter‐pandemic, a bespoke questionnaire was administered to monitor each group. Descriptive statistics were reported (Questions 1–6) and the CBR template was applied to free‐field responses (Questions 7–8). Stage 3: post‐pandemic, a quality of life (QoL) questionnaire was administered to 21 caregivers of children with disabilities and a control group of 11 parents of typically developing children in the same geographical area and 8 caregivers pre‐ and post‐pandemic. Descriptive statistics were applied.

**Results:**

Pre‐pandemic, there was food security, medicine availability, school attendance, social connections and livelihood. Group plans involved livelihood and social inclusion developments. However, member commitment, community attitudes and environmental conditions were ongoing challenges. Inter‐pandemic, some socially distanced group meetings focus on COVID‐19 prevention, livelihood and social support. Livelihood activities were affected variously with reported difficulties including food insecurity, school closures and reduced meeting frequency. Post‐pandemic comparison between caregivers and a control group revealed overall significantly higher caregiver QoL scores. Pre‐ to post‐pandemic evaluations demonstrated overall significantly improved caregiver QoL.

**Conclusions:**

Despite the changing context brought by the COVID‐19 pandemic, the self‐help groups appear to have afforded some protection against the worst psychosocial and economic effects and helped to sustain the caregivers.

**Summary**

- Self‐help groups provide a vehicle for empowering, supporting and sustaining caregivers of children with disabilities in a changing context.
- Self‐help groups and their members are able to adapt to a changing context and maintain their social connections and livelihood activities appropriately.
- Mutual psychosocial support amongst the membership, group financial savings and collective plans for shared livelihood activities may be critical factors in mitigating the worst effects of adverse conditions such as the COVID‐19 pandemic.
- Belonging to a self‐help group may support the caregivers to develop personal and collective resilience, particularly in adverse conditions, and contribute to a better quality of life.

## 1 Introduction

Raising a child with disabilities in circumstances of limited resources presents many challenges. In sub‐Saharan Africa, caregiving duties are typically carried out by a mother or grandmother who is responsible for the health and wellbeing of the child with disabilities (Zuurmond et al. 2019). The caregiver's role is affected by inadequate information about disability causation and relevant interventions, low family income, scarce support services and poor access at a community level (He et al. 2024). Whilst new understanding of biological causes of disability has been revealed (Bunning et al. 2017), superstitious narratives persist that variously attribute disability to a breach of social conventions and preternatural forces (Paget et al. 2016). Such traditional explanations can give rise to stigma and discrimination, both in the immediate family and in the local community. As a result, many caregivers and their children express feelings of being alone, isolation and helplessness (He et al. 2024).

Self‐help groups provide one approach to addressing the challenges in the lives of caregivers. They are collective, mutually supportive groups of people with similar lived experiences who come together for a shared purpose (Gugerty et al. 2019). Identified within the ‘empowerment’ domain of the World Health Organisation community‐based rehabilitation (CBR) matrix (WHO 2010), there is an emphasis on collaborative development and decision‐making amongst the members. Collective resilience, companionship and peer support, knowledge and understanding of disability, mitigation of the effects of stigma, access to information and practical resources have been reported for groups that bring caregivers together (He et al. 2024). Self‐help group initiatives and, more broadly, peer support groups have been used by different communities in sub‐Saharan Africa, for example, caregivers of children with disabilities in Kenya (Bunning et al. 2020; Gona et al. 2020) and Ghana (Zuurmond et al. 2019), mental health service users in Uganda (Nakimuli‐Mpungu et al. 2017), people with epilepsy in Tanzania (Mmbando et al. 2022) and people living with HIV in Kenya (Kako et al. 2021) and in Nigeria (Verinumbe et al. 2024). A process of empowerment lies at the centre of such groups whereby individuals are encouraged to view themselves as capable of influencing and controlling the daily challenges encountered by their own actions (Zimmerman 1995; Brody et al. 2017). It is through sharing their experiences that the members of a group are connected and establish a sense of belonging (O'Connell et al. 2024).

A changing context may pose threats to self‐help and peer support groups. The COVID‐19 pandemic represented a major challenge to people across the globe, with the first reported cases of COVID‐19 in Africa being in February 2020 (Adepoju 2020). By October 2020, only 4% of COVID‐related deaths were in this region (Sotola et al. 2021), which was probably attributable to limited access to healthcare services (Adugna et al. 2020) and low levels of testing for COVID‐19. McKinney et al. (2020) criticised the relevance of WHO recommendations to a LMIC context, for example, wearing a mask, washing hands and disinfecting surfaces. Barriers to the implementation of some preventative measures included a lack of running water and unclear information about ways to mitigate the risk of infection (Abdullahi et al. 2020).

Individuals living with disabilities were considered to be four times more likely to die from COVID‐19 due to health and triage policies that neglected their particular health and informational needs (Kuper et al. 2020; McKinney et al. 2020). During the critical period of the pandemic, local closure of schools not only interrupted children's education (Abdullahi et al. 2020) but also their access to vital school‐feeding schemes (Zar et al. 2020). Other associated impacts on children with disabilities and their families included reductions in livelihood opportunities and income due to local lockdowns and restrictions on social and trade gatherings (Mbazzi et al. 2022; Samboma 2021); and mental and physical health (Mbazzi et al. 2022).

The aim of the current study was to investigate the sustainability of previously established self‐help groups in Kilifi, Kenya, within a changing context. The groups were originally developed in the period 2015–2018 through a research partnership between the University of East Anglia, United Kingdom, and the Kenya Medical Research Institute (KEMRI). They were set up to improve the quality of life of both caregivers and children with disabilities. Caregivers met regularly and embarked on shared livelihood activities for their mutual benefit. These activities included livestock rearing (goats and chickens), farming (maize, cassava, chilli peppers), making liquid soap, harvesting and selling palm wine, making makuti for thatched roofs and buying food stuffs from a wholesaler for community resale. In addition to livelihood activities, facilitated group discussions on economic empowerment, peer support, health and education of children with disabilities and community inclusion (Bunning et al. 2020; Gona et al. 2020) served to raise understanding and stimulate the sharing of ideas amongst the membership. At the end of the set‐up period (2018), there were 11 self‐help groups with a total membership of 154 caregivers (Gona et al. 2020). Individual group membership ranged from 5–20 (*Mdn* = 14). Reported outcomes included improvements to caregiver agency, increased social support and a reduction in the perceived severity of the child's disability (Bunning et al. 2020). Two underlying mechanisms were identified in relation to these changes: *handling goods and money* and *social ties and support*. The first referred to business ventures and income‐generating activities carried out by the groups which was consistent with the economic gains highlighted in Brody et al.’s (2017) systematic review. The second captured the socially unifying nature of self‐help groups as a counterpoint to the isolating experience of caregivers in everyday life (Thoits 2011).

The current study was conducted from 2018 to 2023 focusing on group sustainability. The primary research question was how do self‐help groups function within a changing context? A subsidiary question was how does self‐help group membership serve to sustain caregivers in adverse conditions such as the COVID‐19 pandemic?

## 2 Methods

### 2.1 Design and Setting

An integrated framework of action research and evaluation using mixed methods was adopted (Rademaker and Polush 2022) to assess the sustainability of 11 self‐help groups in Kilifi, Kenya, during which there was the COVID‐19 pandemic (2018–2023). As proposed by Ivankova and Wingo (2018), principles of systematic inquiry were followed in terms of identifying the research problem, forming research questions and defining data collection methods to assess the value of the self‐help group programme. This was conducted over three stages: Stage 1: pre‐pandemic—focus group discussions; Stage 2: inter‐pandemic—administration of a bespoke questionnaire to an official of each group; Stage 3: post‐pandemic—Quality of Life questionnaire to caregivers and a control group.

Prior to the pandemic, the self‐help groups met regularly (at weekly or fortnightly intervals), carried out livelihood activities for their mutual benefit and received visits from the Kenyan‐based project co‐ordinator (J.K.G.) who facilitated group discussions on their approaches to economic empowerment, peer support, health and education of children with disabilities and community inclusion (Gona et al. 2024). During the pandemic, the banning of all social gatherings and closure of schools were made effective from 25 March 2020 and remained largely in place for 12 months. Mass vaccination against COVID‐19 commenced in March 2021, which saw the relaxation of restrictions.

The self‐help groups were located in Kilifi County (2019 census: area= 12 539.7 km^2^; poverty level = 48.4%) (HURU map 2019), a rural area of Kenya with a population of 1 607 000 (2024: www.statskenya.co.ke). It is bordered to the East by the Indian Ocean. Kilifi residents are mainly from the tribes of the Mijikenda (about 80%) and speak mainly Swahili and Giriama. It is one of the poorest areas in Kenya. The majority of Kilifi residents live in dwellings of mud construction consisting of one or two rooms, with no power supply or running water. Families are largely dependent on subsistence farming for income. Pre‐COVID, per capita, the average income (in a typical family of parents and six children) was KES1000 per month—less than $13 (KIHBS, 2015–16). A county‐wide survey of neurological impairment in children 6–9 years revealed a 6.1% prevalence for moderate–severe difficulties with epilepsy, cognition, hearing, motor functioning and vison being the most commonly affected domains (Munga'la‐Odera et al. 2006). This prevalence figure is reflected in the Kenyan national survey of health characteristics for the population, which reported that 6% of females and 5% of males aged 5 and above experienced severe difficulties in everyday functioning (KNBS 2023).

### 2.2 Ethics

The study was approved by the UK University of East Anglia ethics committee (UEA: ETH2122‐0225) in two stages to accommodate changes to the research plan due to the pandemic and by Pwani University, Kenya (Pwani: ERC/PU‐STAFF/002/2022). Information regarding the project was given both orally and in writing to the participants. Consent was obtained by signature or thumbprint. Data were anonymised and stored on a secure server with access granted only to members of the research team.

### 2.3 Study Population

**FIGURE 1:**
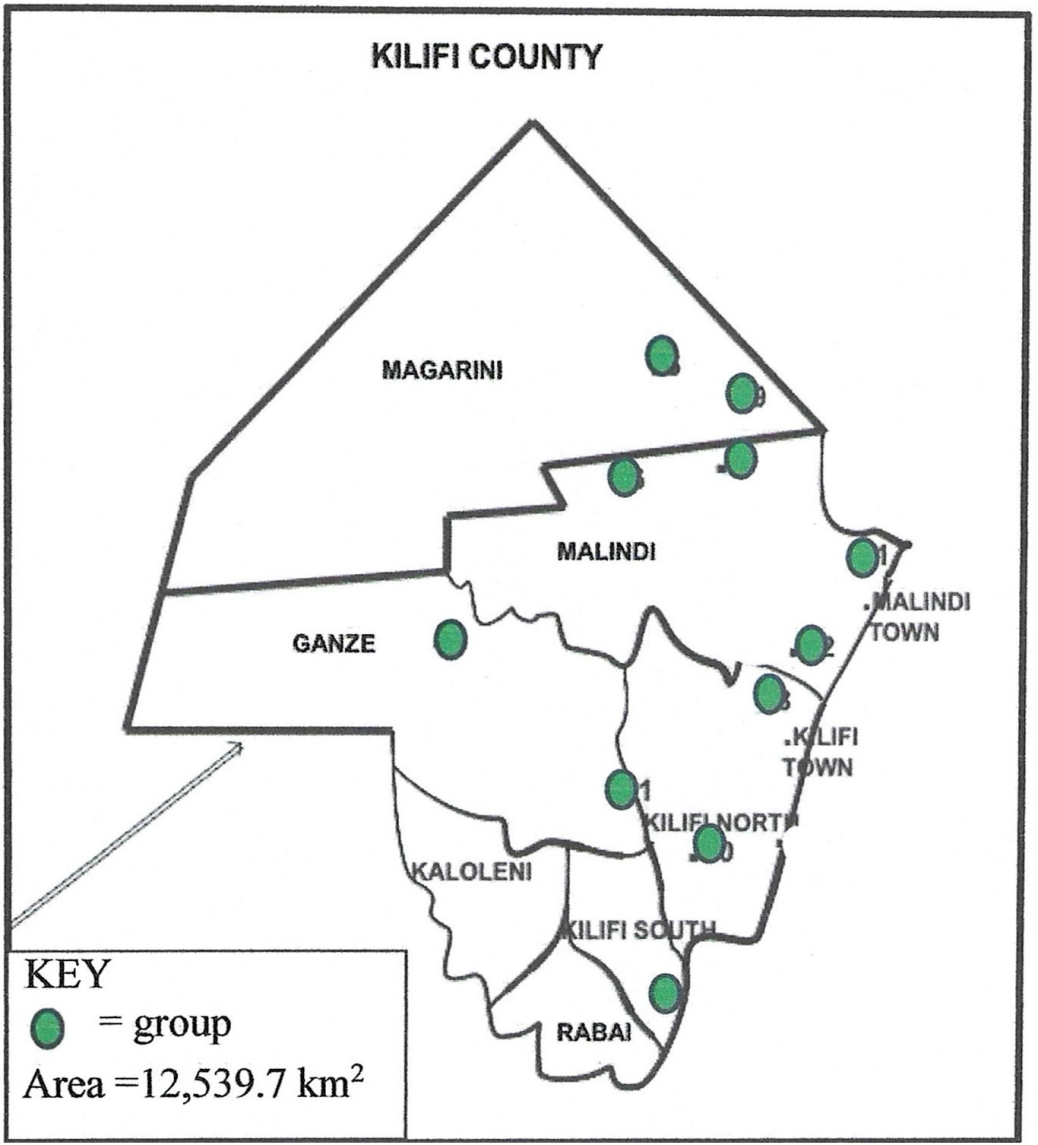
Map of Kilifi County with location of self‐help groups indicated.

The study focused on 11 self‐help groups attended by 154 caregivers of children with disabilities over a 4‐year period. Figure 1 shows a map of the relative locations of the groups in Kilifi County with area in square kilometres indicated.

Only groups that met regularly (e.g., at weekly or fortnightly intervals) and were contactable via a group officer (e.g., chair, treasurer or secretary) were included in the inter‐pandemic phase of the study.

#### 2.3.1 Stage 1: Pre‐pandemic

Forty‐two caregivers across three self‐help groups participated in the focus group discussions (October 2019–January 2020). Originally, the plan was to carryout focus groups with each of the 11 groups across 2 years. However, government restrictions on community gatherings in 2020 prohibited further data collection. As shown in Table 1, out of the 42 caregivers in the three groups, 50% were over 40 years of age. Although the three groups were largely of female composition, two of the groups had male representation (Group 6: 14 females:1 male—a father; Group 9: 10 females:2 males—a father and a man with a disability who was the chairperson). The majority of the caregivers identified as being married (57%); 50% had 3–6 children, 31% had 7–11 children and 7% had more than 11 children. Educational attainment varied across the caregivers with 55% having received no formal education, 21% having completed primary education and 19% achieving only partial completion at primary level. Only two caregivers had completed secondary education. The majority of the caregivers had only 1 child with disabilities (95%) with two caregivers having 2 children with disabilities (5%). Quality of life indicators revealed that 57% lived in a mud and thatch dwelling in poor condition, although 36% had a dwelling with an iron roof and 1 caregiver had a permanent concrete dwelling. The number of meals served per day varied across the sample: 1 meal (24%); 2 meals (50%); 3 meals (21%); and 4 meals (5%). Livestock owned by the caregivers revealed that 62% looked after chickens, 40% reared goats, 21% ducks and 19% had a dairy cow. Group livelihood activities were varied and changed in intensity over time. Group 6 engaged in farming, goat rearing, buying and selling fuel. Group 5 engaged in farming. Group 9 made liquid soap and reared poultry.

**TABLE 1:** Prepandemic focus group discussions: self‐help group characteristics.

SHG ID	No. of members (F:M)	Frequency	Livelihood	No. in focus group discussion
5	15 (15:0)	Weekly	Farming on leased land (2 acres)	7
6	15 (14:1)	Weekly	Rearing goats, selling petrol	10
9	12 (10:2)	Weekly	Making liquid soap for selling, poultry and farming	8

#### 2.3.2 Stage 2: Inter‐pandemic (COVID‐19 Pandemic of 2020–2021)

The study population consisted of any of the 11 self‐help groups who were contactable via one of the group officers (chair, treasurer or secretary). Nine out of the 11 groups responded to the monitoring questionnaire during the monitoring period but with variable consistency.

#### 2.3.3 Stage 3: Post‐pandemic (2021–2022)

Caregivers participated in a Quality of Life assessment in two different comparisons. The first comparison was between two convenience samples: caregivers (*n* = 21) from 6 self‐help groups (19 females:2 males; mean age: 56.5; age range: 37–70) and a control group of parents with typically functioning children (*n* = 11) from the same geographical area (7 females:4 males; mean age: 51.1; age range: 35–78). The control group was recruited by the second author (J.K.G.), a resident of Kilifi who used his local knowledge and contacts to invite members of the community to complete questionnaires. The second comparison was within group pre‐ and post‐pandemic. It involved a small convenience sample of female caregivers (*n* = 8) recruited from three self‐help groups (age: range = 42–70; mean = 53.5; years of caregiving: range = 10–20; mean = 14) who completed pre‐ and post‐pandemic questionnaires.

### 2.4 Data Collection and Analysis

#### 2.4.1 Stage1: Pre‐pandemic

A topic guide with questioning route was developed for use in focus group discussions. The aim was to encourage the caregivers to reflect on their group, any activities undertaken, community engagement and to consider any benefits, challenges and plans for the future. J.K.G., a native of Kilifi and conversant in the local languages who was known to the groups, conducted the focus group discussions, which were audio‐recorded on a portable device. Each focus group was formed of the particular group's membership. The discussions were transcribed in the local language before translation into English. Template analysis (Brooks et al. 2015) was conducted using a framework of a priori themes reflecting the five domains of the CBR matrix (WHO 2010): health, education, livelihood, social and empowerment.

#### 2.4.2 Stage 2: Inter‐pandemic

In response to the spread of COVID‐19 across the African continent, the Kenyan government introduced measures to control the spread of infection. Restrictions on social distancing and county‐wide lockdowns effectively prohibited community gatherings (e.g., meetings, local markets, schools). To monitor the groups during this period, a bespoke questionnaire was devised. Questions 1–6 focused on contact between the members, impacts of COVID‐19 on health of the caregiver and children with disabilities and continuity of group livelihood activities. Response options were read out to the key informant for selection and recording. Questions 7–8 were open‐ended and asked about the challenges faced by the group and any issues concerning families and children with disabilities. Informant responses were recorded in writing in context. J.K.G. administered the questionnaire (versions in Swahili and Giriama) in socially distanced meetings with one of the officials of each group (e.g., chairperson, treasurer or secretary) in an outdoor setting. From April 2020 to July 2021, nine groups completed 56 questionnaires (per self‐help group: mean = 5.09; range = 1–13), with two groups being uncontactable. The number of questionnaires completed each month ranged from 2 to 7 (mean = 3.5).

The data arising from Questions 1–6 were summarised using descriptive statistics. Data coding of the fieldnotes recorded for Questions 7 and 8 used the previously established template of a priori themes (Brooks et al. 2015).

In order to manage bias in the template analysis of focus group discussions (Stage 1: pre‐pandemic) and questionnaire free‐field responses (Stage 2: interpandemic), the data were analysed independently by two pairs of researchers. The transcripts were reviewed and points of questionable clarity were returned to the second author, J.K.G., for checking and back‐translation before analysis could proceed. The separate analyses were compared in a shared review facilitated by the lead author, K.B. Any variations were identified and discussed towards a final consensus.

#### 2.4.3 Stage 3: Post‐pandemic

In order to assess the effects of the COVID‐19 pandemic on the self‐help groups, the Adult Carer Quality of Life Questionnaire (AC‐QoL) (Elwick et al. 2010) was used. The questionnaire explores quality of life in eight domains: support for caring, caring choice, caring stress, money matters, personal growth, sense of value, ability to care and carer satisfaction. Each domain comprises five questions with an accompanying semantic rating scale (never, some of the time, a lot of the time, always) that converts to numeric scores. The higher the overall score, the higher the perceived quality of life. A Swahili version of the questionnaire was administered in person by the second author J.K.G. The questionnaire was used in two comparisons of quality of life: (i) caregivers of children with disabilities who were members of self‐help groups and a control group parents from the same county location but without a child with disabilities postpandemic (February–June 2022); (ii) a small opportunistic sample of 8 caregivers pre‐ (January–March 2019) and post‐pandemic (February–June 2022). Descriptive statistics were applied to both data sets. The nonparametric statistical tests were (i) Mann–Whitney on comparison study data (ii) and Friedman's two‐way analysis on pre‐ and post‐pandemic data with Wilcoxon's signed rank test to investigate domain differences at two time points.

## 3 Results

### 3.1 Stage 1: Pre‐pandemic

The five a priori themes (health, education, livelihood, social, empowerment) were variously associated with positive changes and outcomes affecting the group, the caregivers and their children with disabilities. In addition, there was appraisal of challenges encountered, mutual decisions and plans for the future.

Key: SHG = self‐help group; p = participant.

#### 3.1.1 Health

The caregivers talked of the improved availability of resources to meet the needs of their children with disabilities and their families. They spoke of having enough money for food, medicines and skin care oils for their children, which was attributed to their financial income.

… .. even when the child needs something like body oil or food I have some money to buy. (SHG6/p5)

However, a lack of clean running water was recognised to be a persistent problem encountered by the caregivers.

#### 3.1.2 Education

The caregivers made brief references to their children with disabilities attending school.

#### 3.1.3 Livelihood

Clear benefits of the group livelihood activities were identified. Reference was made to bank savings from income‐generating activities and the group's various business ventures, which included livestock rearing and farming.

We are saving money in the bank. (SHG6/p2)

Each member keeps his own chickens. (SHG9/p1)

However, ever‐present challenges were also identified, in particular, the threat of environmental conditions, with caregivers talking of flooding to farmland and avian flu affecting poultry.

Our shamba (small holding) at the riverbanks is now covered with sand soil. The fertile soil was washed away. (SHG5/p2)

Cognisant of the devastating impacts of drought and floods, the members spoke of their ideas to mitigate the effects of adverse environmental conditions by focusing on livelihoods that were not dependent on a reliable water supply, such as the purchase of equipment to hire out for community events.

We are thinking of buying plates and sufurias (large cooking pots) for hire … it does not depend on the rains. (SHG5/p4)

This demonstrated the group's collective ability to analyse a threat to their livelihood and to generate ideas to circumvent the defined threat.

#### 3.1.4 Social

The caregivers appeared to value the contact amongst group members. They maintained contact with each other through mutual visits to homesteads and the use of mobile phones.

We visit each other. We call each other for those who have mobile phones. (SHG9/p5)

#### 3.1.5 Empowerment

Examples of the empowering practices of the groups were shared, with one group describing how their chairperson had a disability and talked to church congregations to raise awareness and another recounting how they built a house for a caregiver's disabled son. The caregivers suggested ways to promote acceptance of disability through ‘come (ing) up with songs’ (SHG5), having a vehicle displaying a slogan to promote their work and making their children with disabilities more visible.

We should not hide them and making them be seen by people around us. People should see what they can do and what they cannot do. (SHG 6/P3)

### 3.2 Stage 2: Inter‐pandemic

The COVID‐19 pandemic brought about in‐country and locally managed ‘lockdowns’. For the groups, this meant a temporary end to their meetings, access to markets for selling produce, within county travel and school attendance for the children. Despite these challenges, the groups articulated their plans for mitigating the worst effects of the pandemic. Table 2 summarises the group responses to questions about contact and communication amongst the members during the COVID‐19 pandemic, the health of the members and their children with disabilities and the continuation of their livelihood activities.

**TABLE 2:** Interpandemic: Monitoring questionnaire (April 2020 to July 2021: Questions 1–6).

Questions 1–6	Summary of group responses	
	*n*	%
1. Contact amongst members	55	98
2. Communication methods		
a. Social distance in person	49	89
b. Mobile phone contact	6	11
3. SHG members in contact with 3 or more persons	52	95
4. Caregivers with COVID‐19	0	0
5. Children with disabilities with COVID‐19	2	4
6. Continuation of livelihood activities		
a. Yes	14	25
b. Partly	36	64
c. No	6	11

Of the groups who responded to the questionnaire, the majority maintained contact amongst the membership (98% of 55 completed questionnaires), mainly via brief socially‐distanced meetings (*n* = 49, 89%), with some mobile phone communications towards the start of the monitoring period (*n* = 6, 11%). Only one group reported no contact in 1 month. There was report of two children with disabilities being infected by COVID‐19 but no caregivers. Continuity of livelihood activities appeared to be variously affected, with a partial impact being declared mostly (*n* = 36, 64%).

Responses to the free‐field questions captured any challenges encountered, group ideas and action plans.

#### 3.2.1 Health

The groups understood the potential impacts of the changing context on their health and wellbeing with anticipated food insecurity. Since many of the caregivers relied on local markets for selling their own produce and purchasing for their families' needs, there was concern for how they could continue to feed their families under such conditions.

The situation is not good. If things don't change soon, there will be starvation among our children. (SHG2; April 2020)

Early on the groups showed awareness of the relevant measures to prevent the spread of COVID‐19. They talked of wearing face masks and were concerned at the lack of face masks in some cases. The members articulated their actions and plans to keep themselves and their families safe and well.

I insist on washing hands and keeping distance. (SHG9: April 2020)

#### 3.2.2 Education

School closures as part of lockdown measures were a source of stress to the groups, requiring the members to manage their roles as care providers along with domestic chores and livelihood activities. Out of the five domains of the CBR matrix, education appeared to be the one most outside the control of the groups.

Schools not opened giving parents a headache. (SHG8: Dec 2020)

#### 3.2.3 Livelihood

Responses to the free‐field questions were dominated by references to livelihood. The respondents talked of restrictions due to government‐imposed lockdowns, which include closure of local markets. Attempts to maintain group livelihood projects were thwarted by other factors at different times, such as a lack of rain, flooding, nonattendance of a group's chairperson and the departure of members who had taken loans from the group without repaying them.

Some members leave the group with loans; this gives difficulties to the remaining members in paying back the loan given to the group. (SHG8: Apr 2021)

However, plans for income generation were still evident. One group planned to expand their successful flour‐selling business by applying for microfinance and another group planned a poultry project. The groups assessed their current situation and looked to build their livelihoods.

We have constructed a poultry house. We have also got another shamba. We are now looking for a tractor to plough then we plant maize. (SHG8: May 2020)

#### 3.2.4 Social

Some groups continued to meet, but less frequently, whilst others discontinued their meetings temporarily. For face‐to‐face meetings, the members talked of maintaining a social distance. Where SHG meetings were discontinued, the idea of caregivers visiting each other was mooted.

We can't meet as a group. Thinking of visiting individual members and see how we can assist each other. (SHG2: Apr 2020)

#### 3.2.5 Empowerment

Some of the groups were active in advocating for the needs of their most vulnerable members, with representations being made to the local chief's office for food relief. One group said they were not in urgent need of food and preferred it should go to others with a greater need. Plans were articulated to open a bank account and help each other out in farming their shambas (small holdings). Despite the departure of the majority of its members during the COVID‐19 pandemic, the two remaining members of SHG7 expressed their desire to rebuild the group by looking for new members.

### 3.3 Stage 3: Post‐pandemic

As shown in Table 3, a higher quality of life using the AC‐QoL (Elwick et al. 2010) was reported for caregivers compared to the control group, with significant differences between the groups for the following four domains: ‘support for caring’, ‘money matters’, ‘personal growth’ and ‘ability to care’. Any differences on the remainder of the domains (i.e., ‘caring choice’, ‘caring stress’, ‘sense of value’, ‘carer satisfaction’) were nonsignificant.

**TABLE 3:** Postpandemic comparison of AC‐QoL domain scores: caregivers and control group.

Domain	Test score (Mann–Whitney: *p* < 0.05)*	Caregivers		Control	
		Mdn	Variance	Mdn	Variance
Support for caring	< 0.001*	5.0	8.348	3.0	3.618
Caring choice	< 0.271	11.0	25.962	10.0	17.491
Caring stress	< 0.168	15.0	25.529	10.0	14.818
Money matters	< 0.025*	6.0	10.562	3.0	3.794
Personal growth	< 0.001*	10.0	4.090	9.0	2.473
Sense of value	< 0.238	11.0	11.490	10.0	4.0
Ability to care	< 0.016*	10.0	11.757	6.0	11.964
Carer satisfaction	< 0.074	13.0	4.157	11.0	4.618

Pre‐ to postpandemic AC‐QoL scores for the caregivers (*n* = 8) revealed a significant increase in the overall perceived quality of life of the caregivers (X ^2^ = 45.583; *p* < 0.001; df = 7). As shown in Table 4, gains were reported for all the domains, with statistically significant improvements in three domains: ‘support for caring’, ‘caring stress’ and ‘ability to care’.

**TABLE 4:** Caregiver Ac‐QoL: pre‐ to postpandemic comparison.

Domain	Test scores (Wilcoxon matched pairs *p* < 0.05)*	Pre		Post	
		Mdn	Variance	Mdn	Variance
Support for caring	< 0.043*	5.0	5.839	6.5	6.268
Caring choice	< 0.248	6.0	7.696	12.5	23.839
Caring stress	< 0.021*	7.5	18.5	15.0	28.125
Money matters	< 0.061	3.5	16.125	6.0	11.696
Personal growth	< 0.220	10.0	10.5	12.0	4.696
Sense of value	< 0.885	10.5	16.214	12.0	10.982
Ability to care	< 0.012*	4.5	28.0	10.5	11.643
Carer satisfaction	< 0.43	12.0	5.696	13.0	3.643

## 4 Discussion

Pre‐pandemic, the three self‐help groups who participated in focus group discussions appeared to be thriving in terms of livelihood, health and education, social connections and caregiver empowerment. The caregivers recognised the benefits of income generation associated with their livelihood activities, which included money for food and medicines to support the health and wellbeing of their children with disabilities, their families and themselves, as well as savings. Despite environmental challenges, for example, a lack of clean running water and threats to livelihood activities, such as avian flu affecting poultry‐rearing and floods or drought conditions affecting farming, the group members continued to make plans for growing their businesses, making social connections with the community and future group initiatives. Caregiver empowerment was evident in their talk of diversifying livelihood activities to mitigate the worst effects of drought and floods on farming in particular. Furthermore, the caregivers considered possible ways of facilitating the visible presence of their children to stimulate community acceptance.

Inter‐pandemic, government lockdowns compounded the challenges of already present drought conditions in East Africa. Two groups disbanded during this period and there was no further contact. For the remaining nine groups, however, contact amongst the members and livelihood activities were only partially impacted, with meetings occurring less frequently and livelihoods affected variously by closed markets and restricted travel. However, plans to progress the groups continued and these were largely focused on pursuing livelihood projects, following government advice to prevent the spread of COVID‐19, providing support to each other and progressing the group's standing in the community.

Post‐pandemic measures of quality of life (AC‐QoL: Elwick et al. 2010) revealed that the caregivers had a significantly higher quality of life compared to the control group. Significant differences between the groups were reported in four domains where the caregiver group achieved higher scores: ‘support for caring’ (perceptions of practical, emotional and professional support received), ‘money matters’ (assessment of personal financial situation), ‘personal growth’ (view of own skills and development as a caregiver) and ‘ability to care’ (feelings of own competency in carrying out the caregiving role). Pre‐ to post‐pandemic changes in caregiver quality of life showed maintenance or small gains in five of the eight domains, with significant growth in the three domains of ‘support for caring’, ‘caring stress’ (psychological and physical) and ‘ability to care’.

### 4.1 Changing Context

Up until the onset of the COVID‐19 pandemic, income generation was a major focus affecting both livelihood progression and also the professed goals of the membership. Financial income corresponded to food security for the family and was also associated with care of the child with disabilities. This corresponds to the foundation of Maslow's hierarchy of human needs (1943), physiological, which prioritises food, water and shelter, although not exclusively (Koltko‐Rivera 2006). The challenges of living in a low‐income setting and performing the role of caregiver to children with disabilities were ongoing, with environmental factors an ever‐present threat. Pre‐pandemic, the changing climatic context appeared to be addressed by the problem‐solving ability of the groups with livelihood proposals that did not depend on the rains. This resonates findings by Brody et al. (2017) who identified the growing capabilities of self‐help group members in addressing challenges that presented themselves. However, the effects of the COVID‐19 pandemic on group livelihoods were a major source of stress to caregivers as local restrictions affected all forms of social gatherings, including attendance at markets for selling produce (Mbazzi et al. 2022; Samboma 2021).

Pre‐pandemic, the word ‘group’ was used frequently in focus group discussions, conveying a sense of belonging amongst the membership, which is consistent with O'Connell et al. (2024) and Gugerty et al. (2019). Meeting the needs of their children with disabilities was a shared concern for the caregivers as they spoke of the benefits experienced by their children with disabilities in relation to health, education and livelihood (Bunning et al. 2020). One group ascribed positive value to their chairperson, a young man with a physical disability, and talked of his efforts in reaching out to the local community. However, the financial pressures of curtailed livelihoods during the pandemic threatened the stability of the membership and in some cases led to the departure of members who had taken loans from the group. In this respect, the financial pressures of the changing context *did* affect the membership of some groups. Nevertheless, the caregivers viewed their financial situation (i.e., ‘money matters’) as significantly better than the control group, possible helped by having shared savings to draw on midst restricted livelihood activities.

More generally, the usual operation of self‐help groups, with weekly meetings for business and social connectivity, was disrupted. However, communications amongst the members continued through socially distanced meetings in the outdoors and/or mobile phone contact. The forced restrictions meant that the children could no longer attend school and livelihood activities were curtailed or stopped completely. Once country‐wide lockdowns were introduced, caregiver concerns shifted to food security for their families (Samboma 2021), thereby defaulting to the first level of human need: physiological (Koltko‐Rivera 2006; Maslow 1943). In these circumstances, the caregivers recognised the needs of others in their own group, as well as in other groups, most frequently in relation to food supply. The intrapersonal construct defined in Zimmerman's (1995) empowerment theory was evidenced in caregiver self‐awareness of their own capacities to initiate change by recommending that food relief should go to those in greatest need.

Only two children with disabilities were reported to be infected with COVID‐19, no caregivers and no deaths, which is consistent with report from other countries on the African continent (Sotola et al. 2021). The groups were aware of preventative measures to limit the spread of COVID‐19, including cancellation of face‐to‐face meetings or at least reduced participant numbers and use of telephone communication. Remarks about the need for social distancing and the wearing of face masks demonstrated caregiver understanding of the need to prevent transmission, which is counter to Abdullahi et al.'s (2020) observations. It is possible that the structure of the self‐help groups facilitated the sharing of critical information amongst the members (Verinumbe et al. 2024). This in turn may have helped to maintain their health during the pandemic, together with the fact that their meetings typically occurred in outside venues, which presented a lower risk of transmission.

Livelihood projects were not only affected by the COVID‐19 restrictions but also by other factors, such as a lack of rains, flooding, nonattendance of a group's chairperson and the departure of members. Thus, the impact of COVID‐19 was viewed in the context of ongoing environmental and human factors. Despite these challenges, group planning discussions for their livelihood activities continued. Membership of a collective enterprise may have strengthened the resilience of group members in the face of livelihood stresses (He et al. 2024; Mmbando et al. 2022; Nakimuli‐Mpungu et al. 2017; Verinumbe et al. 2024; Zuurmond et al. 2019).

### 4.2 Sustainability

The higher levels of caregiver perceived quality of life compared to the control group, post‐pandemic, might be attributable to the ‘safety net’ afforded by membership of a self‐help group. Certainly, the groups had bank savings that would have sustained them during the lengthy lockdowns when livelihood activities were curtailed to lesser or greater extents. However, caregiver sense of belonging to a self‐help group and the established relationships amongst the members may have represented a buffer to the impacts of social restrictions (Gugerty et al. 2019; O'Connell et al. 2024). ‘Support for caring and ‘ability to care’, which reflect caregiver perceptions of practical and emotional support and self‐knowledge of their skills and coping ability respectively, were significantly higher for the caregivers in comparison with the control group, as well as in the pre‐ to post‐pandemic measures. Despite school closures, reduced income and ban on social gatherings, the shared experience of bringing up a child with disabilities provided mutual affirmation of caring skills amongst the caregivers (Gugerty et al. 2019; O'Connell et al. 2024). Furthermore, caregiver ‘personal growth’ associated with positive caring experiences was significantly higher than for the control group, which is consistent with He et al. (2024). At the start of the pandemic in 2020, the self‐help groups had been functioning for around 4 years (Bunning et al. 2020; Gona et al. 2020) during which their collaborative enterprises, interpersonal relationships and collective problem‐solving abilities had been established. Thus, they had skills and practices to draw on in the face of new challenges which is consistent with findings reported in other self‐help/peer support group initiatives (e.g., Mmbando et al. 2022; Nakimuli‐Mpungu et al. 2017; Kako et al. 2021; Verinumbe et al. 2024; Zuurmond et al. 2019).

Improvements in ‘caring stress’ levels pre‐ to postpandemic indicates that the pandemic did not negatively affect the mental and physical stresses experienced by the caregivers. It is possible that the caregiver's confidence in their own caring abilities served to reduce any mental and physical strain. Improved ‘support for caring’ might be attributable to the ongoing monitoring activities, such as mobile phone contact between the members and from the project manager (J.K.G.), albeit in a changed way, that helped to maintain group members (O'Connell et al. 2024; Gugerty et al. 2019; He et al. 2024).

### 4.3 Limitations

Demographic data collected on the self‐help group members did not differentiate between grandmothers and mothers acting as caregivers to the children with disabilities. This would be useful information in future research. Up until the COVID‐19 pandemic, only three out of 10 potential focus group discussions were completed. During the COVID‐19 pandemic, the rural distribution of the self‐help groups and caregivers and local restrictions prohibited further focus group discussions and the completion of a validity check on captured data with each participating group. Whilst the focus group discussions provide some useful illustrations of self‐help group developments, they represent only three units of analysis. In response to the restrictions brought about by government‐directed lockdowns, we revised our plans and developed a bespoke monitoring questionnaire that could be administered to representatives of each group. Time constraints meant the questionnaire was not piloted prior to its use. The completeness of the data set was affected by local COVID‐19 restrictions (Groups 1 and 3) and internal disruptions (Groups 4, 7, 10 and 11). A small convenience sample completed both pre‐ and post‐pandemic measures. Although a larger sample was recruited pre‐pandemic to complete the AC‐QoL (Elwick et al. 2010), post‐pandemic measures were only completed by eight of the original sample, the remainder being out of contact with their groups at the time of data collection. Extension to the data collection period in the post‐pandemic phase may have helped access to the remainder of the participants. It is possible that the caregivers who were accessible were most committed to the groups (e.g., regular attendees of meetings) thereby introducing potential bias to the sample. Future research should consider recruiting a random sample of caregivers to eliminate the possibility of bias. The post‐pandemic comparison between caregivers and control group members used convenience samples. The control group involved in the assessment was only broadly matched in terms of geographical setting, mean age and family size due to time constraints in the project's life. Future research should aim to match participants on the basis of age, gender, final level of educational attainment, marriage status, number of children in the family and poverty‐level indicators (e.g., dwelling characteristics, meals served per day, livestock ownership).

## 5 Conclusions

Self‐helps groups provide an effective mechanism for caregivers of children with disabilities in a changing context. Through their participation in self‐help groups over time, caregivers develop a sense of agency. They are empowered to take control of their situation and to take actions that improve their financial situation, their sense of belonging to a community and their collective problem‐solving ability. Adverse conditions such as the COVID‐19 pandemic pose a threat to group stability and progress. However, the self‐help groups themselves facilitate caregivers to maintain contact with each other, engage in mutual support, whilst also planning for the future. For the caregivers themselves, group membership supports their growing personal and collective resilience as demonstrated in their self‐belief in their abilities as carers, awareness of the support around them and improvements in emotional and physical stress levels. Self‐help groups have the potential to mitigate the worst effects of a changing context on its members, as in the case of the COVID‐19 pandemic.

COVID‐19 pandemic excepted, the context for self‐help groups in a low‐income setting is one of continuous change affected by extreme environmental conditions. Research in these circumstances needs to be responsive, rigorous and adaptable. Use of an integrated framework of action research that embraces both qualitative and quantitative methods offers possibilities for evaluating the worth of such a programme as self‐help groups. In this way, practice may be evaluated and problems addressed as they occur.

Finally, self‐help groups have been shown to function effectively within a changing context, to sustain the caregiver members by affording them some protection in extreme adverse conditions and ultimately empowering them such that they are stronger together.

## Author Contributions

**K. Bunning:** conceptualization, investigation, funding acquisition, writing – original draft, methodology, validation, visualization, writing – review and editing, formal analysis, project administration, data curation, supervision, resources, software. **J. K. Gona:** conceptualization, investigation, writing – review and editing, methodology, validation. **S. W. Wanjala:** writing – review and editing, project administration. **S. Hartley:** conceptualization, writing – review and editing, methodology.

## Ethics Statement

The study was approved by UEA (ETH2122‐0225) and Pwani (ERC/PU‐STAFF/002/2022).

## Conflicts of Interest

The authors declare no conflicts of interest.

## Data Availability

The data that support the findings of this study are available on request from the corresponding author. The data are not publicly available due to privacy or ethical restrictions.
